# The Possible Significance of Proteomics in Understanding Molecular Mechanisms of Progressive Supranuclear Palsy, Corticobasal Degeneration, Multiple System Atrophy, and Dementia with Lewy Bodies

**DOI:** 10.3390/cells15090759

**Published:** 2026-04-23

**Authors:** Natalia Madetko-Alster, Dagmara Otto-Ślusarczyk, Marta Struga, Piotr Alster

**Affiliations:** 1 Department of Neurology, Medical University of Warsaw, 03-242 Warszawa, Poland; 2 Department of Biochemistry, Medical University of Warsaw, 02-091 Warszawa, Poland; dagmara.otto@wum.edu.pl (D.O.-Ś.); marta.struga@wum.edu.pl (M.S.)

**Keywords:** atypical parkinsonisms, progressive supranuclear palsy, dementia with Lewy bodies, proteomics

## Abstract

Atypical Parkinsonisms are a diverse group of diseases associated with multiple pathologies, including synucleinopathies and tauopathies. Atypical Parkinsonisms include progressive supranuclear palsy, corticobasal degeneration, multiple system atrophy, and dementia with Lewy bodies. The examination of these diseases is complicated due to their overlapping clinical manifestations. Hence, tools enabling reliable supplementary assessment of atypical Parkinsonisms are needed. The most common methods involve neuroimaging; however, these evaluations generally involve basic magnetic resonance imaging and indicate possible morphological changes. Less attention is given to disease background assessment. Biochemical assessment enables a more detailed examination of the factors impacting neurodegenerative processes. The features that may impact the pathophysiology of these diseases include metabolic abnormalities, excessive inflammation, and environmental factors. In this context, proteomic evaluation, as analyzed in this article, could partly address the insufficiently described aspects of the unclear pathological mechanisms related to atypical Parkinsonisms.

## 1. Introduction

Atypical Parkinsonisms are a group of diseases associated with diverse pathologies, mostly tauopathies and synucleinopathies. Tauopathies include progressive supranuclear palsy (PSP) and corticobasal degeneration (CBD), and synucleinopathies include dementia with Lewy bodies (LBDs) and multiple system atrophy (MSA) [1,2,3,4]. There are some pathophysiological discrepancies between these conditions. The link between neuroinflammation and microglial activation is triggered by pathological cascades that culminate in tau pathology and the clinical presentation of PSP [5]. Pronounced neuroinflammation has been proposed as a key mechanism in the pathogenesis of MSA [6]. However, it remains unclear whether inflammatory processes initiate protein aggregation, arise secondary to neurodegeneration, or exacerbate disease progression [7]. The pathophysiology of corticobasal syndrome (CBS) is atypical among Parkinsonisms and has the least verified background due to its rarity and diverse clinical manifestations [8]. In the case of DLB, an increase in markers of both central (cerebral) and peripheral inflammatory processes is observed in individuals at the mild or prodromal stages of the disease [9].

The diseases vary in their clinical manifestations; however, they are generally associated with poor prognosis, pronounced clinical deterioration, and a life expectancy after diagnosis rarely exceeding 10 years. Additionally, there are discrepancies in the clinical evaluation of PSP regarding subtypes. The two major phenotypes of the disease, progressive supranuclear palsy–Richardson’s syndrome (PSP-RS) and progressive supranuclear palsy–Parkinsonism-predominant (PSP-P), significantly differ in their clinical evolution and symptomatology [1,10]. PSP-RS is associated with significant oculomotor dysfunction, postural disturbances, pronounced deterioration, poor prognosis, and a lack of response to levodopa treatment. PSP-P involves a more gradual evolution and Parkinsonian syndrome, with a partial response to levodopa treatment [1,10]. Definitive diagnoses of atypical Parkinsonisms are made based on neuropathological evaluation only. In vivo examinations enable a clinically established, probable, or possible diagnosis, considering also the diverse pathologies underlying clinical manifestations. In the case of CBS, the most common pathology, CBD, is linked to about half of cases, while the others are associated with other pathologies, including Alzheimer’s disease (AD), frontotemporal dementia (FTD), PSP, etc. Supplementary examinations of atypical Parkinsonisms are mostly based on neuroimaging. Neuroimaging is considered a supportive feature in the diagnostic criteria for PSP, indicating factors such as predominant midbrain atrophy or hypometabolism and postsynaptic striatal dopaminergic degeneration [1]. In CBD, neuroimaging is considered more as a method of assessment indicating other pathologies, rather than a criterion for the disease [2]. The contemporary criteria for MSA indicate MRI markers as abnormalities observed within the putamen and middle cerebellar peduncle. Abnormal cardiac sympathetic imaging is considered an exclusion criterion for MSA [3]. Features associated with the DLB include reduced transporter uptake in the basal ganglia in positron emission tomography and single-photon-emission computed tomography [4]. The pathophysiological background of atypical Parkinsonisms remains insufficiently explored.

A differential diagnosis of atypical Parkinsonian syndromes (APS) in clinical practice is challenging due to the disease’s diversity and overlapping symptomatology, especially in the early stages. The currently in-force diagnostic criteria do not provide methods enabling distinctions between APS manifestations except neuropathological examination. This often leads to misdiagnosis and medical errors involving, e.g., incorrect qualification for advanced therapies reserved for Parkinson’s disease. Therefore, there is a growing need to expand differential diagnostic tools and maximize diagnostic accuracy. Emerging data concerning molecular mechanisms of neurodegeneration indicate the utility of proteomic analysis in this area, which served as a basis for this review.

The literature search was performed using the PubMed database. The authors used the following phrases during the search: “proteomics atypical parkinsonims”, “proteomics PSP”, “proteomics CBD”, “proteomics MSA”, and “proteomics DLB”. Due to the limited number of sources, the authors included papers published between 1995 and 2025.

## 2. Biochemical Evaluation of Atypical Parkinsonisms: General Perspective

Biochemical examination of atypical Parkinsonian syndromes is an evolving method of assessment that is less commonly used in clinical practice. The parameters analyzed may include inflammatory parameters, neurotrophic factors, or neurofilament light chains, associated with the progression and differential diagnosis of diseases and their subtypes [11,12,13].

There is increasing interest in non-specific inflammatory factors based on morphological assessment of blood samples, such as the neutrophil-to-lymphocyte ratio; however, their levels may be impacted by multiple features [14,15]. Nevertheless, their strength lies in their high accessibility.

Non-specific parameters based on blood morphology, neurotrophic factors, or neurofilament chains have limited utility in differential diagnosis due to their low specificity and insufficient data concerning, i.a., cutoff points. Although statistically significant differences are observed when comparing these parameters among large groups of patients, a single measurement is insufficient to enable a differential diagnosis. Moreover, these parameters can be easily altered by multiple processes unrelated to neurodegeneration, e.g., infection, metabolic abnormalities such as diabetes, or drug intake.

These biomarkers should be interpreted as diagnostic indicators as well as indirect reflections of underlying biochemical processes, including neuroinflammation, axonal degeneration, and impaired neuronal homeostasis.

However, these markers provide only a partial insight into disease mechanisms due to their limited specificity. In this context, proteomic approaches offer a more comprehensive and system-level perspective, enabling the identification of coordinated molecular alterations underlying atypical Parkinsonisms.

## 3. Proteomic Evaluation of Atypical Parkinsonisms

The proteomic evaluation of atypical Parkinsonisms is an evolving aspect of examination. A cross-sectional study including 136 participants revealed that up to 12.6% of SomaLogic-developed aptamers (SOMAmers) were differentially expressed in PSP compared with controls [16]. Most SOMAmers revealed a reduced signal. The outcome of the research showed co-expression models linked to PSP-cohorts, including synaptic function/JAK-STAT, vesicle cytoskeletal trafficking, and cytokine–cytokine receptor interaction. Analyses of the cerebrospinal fluid in Parkinson’s disease (PD), MSA, and PSP revealed decreased levels of synaptic protein neuronal pentraxin-2 (NPTX2) [17]. This highlights the significance of synaptic dysfunction in these diseases. The study indicated the possible relevance of mitochondrial ribosome downregulation in PSP. Mass spectrometry-based proteomic assessment of the human globus pallidus of PSP patients showed that abnormalities in the mitochondrial respiratory electron transport chain complex may play a role in the pathogenesis of the disease [18]. The indicated processes could also be caused by neurodegeneration. Another study suggested the possible usefulness of evaluating ATP6AP2, NEFM, and LAMP2 in the differential diagnosis of PSP and PD or healthy controls [19]. Therefore, it may be reasonable to extend the examination of neurofilament and lysosomal membrane markers in disease evaluation. An analysis of neurodegenerative diseases based on a novel multiplex proteomic method, nucleic acid-linked immuno-sandwich assay (NULISA), showed increased levels of serum neurofilament light chain as well as upregulation of inflammatory markers in PSP and FTD [20].

The characteristic proteomic signatures of MSA include the upregulation of aquaporin 4. This astrocytic protein, though linked to beta-amyloid clearance, is extensively studied in terms of its significance in alpha-synuclein clearance. The authors of [17] indicated the possible relevance of cytoplasmic copper chaperones, such as ATOX1, which has been linked to the in vitro anti-aggregatory properties of alpha-synuclein. A study presenting the proteome profiling of cerebrospinal fluid obtained from MSA patients showed increased total fibrinogen levels and immune-related components in the soluble fraction. This observation was also confirmed in other Parkinsonisms, such as PSP and DLB, but not in PD [21]. An evaluation of the proteomic profiles of the patient-derived striatal medium spiny neurons of three MSA cases revealed multidimensional differences in 151 proteins compared with controls [22].

There are insufficient data regarding the proteomic profile of CBD due to its rarity and the difficulties in its examination. A study comparing one CBD case and three brains of non-CBD cases revealed factors linked to up- or downregulation [23]. Two proteins were upregulated in the brains of patients with corticobasal degeneration: protein-L-isoaspartate (D-aspartate) O-methyltransferase and non-muscle cofilin 1. Six proteins were downregulated, including carbonyl reductase [NADPH] 1, two isoforms of peptidyl-prolyl cis-trans isomerase A, ubiquitin carboxyl-terminal hydrolase L1, brain phosphoglycerate mutase 1, and chain A of human peroxiredoxin. Given that the study’s outcome was based on the analysis of one CBD case, the obtained results have limited relevance and should be interpreted as possible factors, encouraging further research on statistically relevant groups of cases. At this stage, reaching unambiguous conclusions is not possible due to insufficient data regarding the proteomic analysis of CBD.

DLB is examined using proteomic analysis. A study evaluating PD, Parkinson’s disease with dementia (PDD), and Alzheimer’s disease (AD) observed differences in TRIM33 and cysteine/glutamate transporter (SLC7A11) throughout the brain regions [24]. Additionally, decreased levels of autophagy protein p62 (sequestosome-1) were detected in PDD and DLB, which distinguished them from AD. In a study evaluating CSF in AD and DLB, the levels of CRH and MMP3 were higher in AD and lower in DLB, whereas DDC and GH showed more pronounced abnormalities in DLB [25].

Overall, these proteomic findings indicate that atypical Parkinsonisms are associated with alterations in a limited number of core cellular systems, including synaptic regulation, mitochondrial energy metabolism, protein quality control, and glial–vascular interactions. These proteins should not be interpreted as isolated biomarkers; their biological relevance emerges only when considered within interconnected biochemical pathways governing neuronal homeostasis.

Therefore, while proteomic studies provide valuable descriptive data, their full significance lies in mechanistic integration. Understanding how these alterations interact at the biochemical level is essential to explain disease-specific pathophysiology and connect molecular findings with clinical phenotypes in atypical Parkinsonisms.

## 4. Biochemical Significance of Proteomic Alterations in Atypical Parkinsonisms

Proteomic studies on atypical Parkinsonisms have identified a recurrent set of proteins that, when biochemically examined, delineate key cellular systems underlying neurodegeneration. This section integrates proteomic findings by highlighting shared and partially overlapping biochemical vulnerabilities, thereby positioning these proteins within interconnected cellular systems that underlie neurodegeneration.

Rather than functioning as isolated biomarkers, these proteins participate in interconnected processes regulating synaptic stability, cytoskeletal integrity, mitochondrial energy metabolism, protein quality control, and glial–vascular homeostasis. Interpreting their roles mechanistically allows proteomic findings to be positioned within a coherent biochemical framework. The principal biochemical functions and mechanistic implications of the proteomic proteins discussed in this section are summarized in Table 1.

Alterations in synapse-associated proteins, most notably neuronal pentraxin-2 (NPTX2), indicate impaired activity-dependent synaptic remodeling. NPTX2 plays a key role in the activity-dependent organization of AMPA receptors at excitatory synapses; therefore, reduced NPTX2 levels may reflect impaired synaptic adaptability rather than mere synapse loss [17,26,27].

At the molecular level, reduced NPTX2 levels may disrupt activity-dependent organization of AMPA receptor complexes, leading to impaired synaptic scaling and reduced adaptability of neuronal networks. This suggests that early synaptic dysfunction may represent a functional stage of neurodegeneration preceding structural neuronal loss, particularly in PSP.

At the cellular level, such impairment may precede structural neurodegeneration by rendering synapses functionally inefficient and metabolically vulnerable, particularly in disorders characterized by early synaptic failure such as PSP [17].

This synaptic vulnerability is closely coupled to cytoskeletal integrity, as reflected by changes in neurofilament proteins.

Neurofilament light and medium chains are essential determinants of axonal caliber and transport efficiency. Their altered levels are best interpreted as a convergent marker of axonal stress, consistent with upstream disturbances in energy metabolism, cytoskeletal dynamics, and protein turnover rather than a disease-specific mechanism [11,19,20].

Synaptic and axonal processes are among the most energy-demanding functions of neurons; therefore, disruption of synaptic and cytoskeletal integrity increases metabolic stress on neuronal mitochondria. Mitochondrial proteins identified in proteomic studies further indicate bioenergetic dysfunction underlying these synaptic and cytoskeletal changes.

Alterations in components of the respiratory electron transport chain and mitochondrial ribosomal proteins suggest impaired coordination between mitochondrial protein synthesis and oxidative phosphorylation. Biochemically, such a mismatch may lead to reduced ATP production and increased reactive oxygen species generation without complete mitochondrial failure, a state particularly detrimental to neurons with high energetic demands and limited metabolic flexibility, as observed in PSP [17,18].

This likely represents a state of partial bioenergetic failure rather than complete mitochondrial dysfunction, leading to reduced cellular reserve and increased susceptibility to additional stressors such as synaptic activity or protein aggregation.

Such bioenergetic imbalance may impair neurons’ ability to sustain high-frequency synaptic transmission, thereby linking mitochondrial dysfunction directly to synaptic failure.

Thus, mitochondrial dysfunction is best understood as an amplifier of synaptic and axonal vulnerability rather than an isolated pathogenic process.

Increased oxidative and metabolic stress may lead to a reduction in mitochondrial functional reserve, thereby limiting neuronal capacity to adapt to additional cellular load. Rather than constituting an independent pathogenic factor, mitochondrial stress is likely to exacerbate synaptic and axonal dysfunction. This subthreshold mitochondrial dysfunction amplifies synaptic and axonal vulnerability, linking energy metabolism to structural neuronal integrity. Because mitochondrial dysfunction directly affects ATP availability and redox homeostasis, it also has important consequences for cellular protein quality control mechanisms.

Defective protein quality control represents a central biochemical mechanism implicated across atypical Parkinsonisms. Proteins involved in autophagy–lysosomal pathways, including lysosome-associated membrane protein 2 (LAMP2) and the autophagy adaptor p62/sequestosome-1, play key roles in the selective removal of ubiquitinated and aggregation-prone substrates. Alterations in the expression of these proteins indicate reduced efficiency of selective autophagy rather than a global impairment of proteolysis.

From a biochemical standpoint, impaired selective autophagy preferentially affects long-lived and aggregation-prone proteins such as tau and α-synuclein, providing a mechanistic link between proteomic alterations and the accumulation of pathological protein aggregates [19,24].

Additionally, impaired protein clearance may interfere with intracellular trafficking and signaling pathways, further contributing to neuronal dysfunction.

Decreased levels of autophagy protein p62 (sequestosome-1) were detected in PDD and DLB, which distinguished them from AD. A study evaluating CSF in AD and DLB revealed higher levels of CRH and MMP3 in AD and lower levels in DLB, whereas DDC and GH showed more pronounced abnormalities in DLB [25].

In CBD, for which proteomic data remain limited due to disease rarity and restricted tissue availability, reported alterations in ubiquitin carboxyl-terminal hydrolase L1 (UCHL1) and protein repair enzymes provide insight into the impaired ubiquitin recycling and reduced capacity for proteome maintenance under conditions of oxidative stress [23]. Although based on sparse datasets, these findings are consistent with broader proteostasis disturbances observed in other atypical Parkinsonian syndromes and support the notion that diminished protein quality control represents a shared biochemical vulnerability rather than a disease-specific anomaly.

While disturbances in protein quality control constitute a shared intracellular vulnerability across atypical Parkinsonisms, additional proteomic signatures further refine disease-specific biochemical landscapes, particularly in DLB. In DLB, additional proteomic alterations involve proteins associated with redox balance, autophagy, and neuroendocrine signaling. Changes observed in the cysteine/glutamate transporter SLC7A11 indicate dysregulation of cellular redox homeostasis, as this transporter regulates cystine uptake required for glutathione synthesis while modulating extracellular glutamate levels. Dysregulation of SLC7A11 may impair cystine uptake required for glutathione synthesis while simultaneously increasing extracellular glutamate levels, thereby linking oxidative stress with excitotoxic mechanisms in synucleinopathies [24].

Reduced levels of the autophagy adaptor p62 (SQSTM1), already implicated in selective autophagic clearance, further support exhaustion of protein quality control mechanisms in DLB and PDD, consistent with impaired removal of aggregation-prone substrates such as α-synuclein [24].

Proteins primarily reflecting neuroendocrine and extracellular matrix-related alterations, including CRH, MMP3, DDC, and GH, distinguish DLB from Alzheimer’s disease at the biochemical level. Altered CRH and GH levels may reflect neuroendocrine involvement, whereas changes in DDC are compatible with broader alterations in catecholamine-related pathways beyond the nigrostriatal system. Differential MMP3 levels may reflect disease-specific modulation of extracellular matrix remodeling and inflammatory signaling [25].

These neuronal and neuroendocrine alterations are embedded within a broader cellular milieu shaped by glial activity and vascular integrity.

Glial- and vascular-associated proteins add an important extracellular dimension to this biochemical network. Upregulation of aquaporin-4 reflects altered astrocytic regulation of water homeostasis and glymphatic clearance, potentially impairing the removal of extracellular metabolites and misfolded proteins and thereby indirectly increasing neuronal stress [17]. Concurrent enrichment of fibrinogen and other immune- or coagulation-related components implicates disruption of the blood–brain barrier and extracellular matrix remodeling. Biochemically, elevated fibrinogen is consistent with blood–brain barrier dysfunction and may contribute to a proinflammatory extracellular milieu, linking vascular pathology with neuroinflammatory amplification in MSA, PSP, and DLB [21,22]. Although atypical Parkinsonisms share common biochemical vulnerabilities—including synaptic dysfunction, mitochondrial impairment, and defective protein quality control—their relative contribution differs across diseases. In PSP, neuronal and mitochondrial dysfunction appear to dominate, consistent with early synaptic failure and high metabolic demand. In MSA, proteomic alterations emphasize glial and vascular components, including aquaporin-4 and fibrinogen-related pathways. In DLB, disturbances in redox balance and excitotoxicity, particularly involving SLC7A11, play a more prominent role. The available data on CBD indicate impairment of ubiquitin-dependent proteostasis.

Overall, the proteins identified in proteomic studies of atypical Parkinsonisms delineate a network of interdependent biochemical systems rather than isolated disease-specific markers. Synaptic adaptability, axonal structure, mitochondrial efficiency, selective autophagy, and glial–vascular regulation form a continuum of cellular homeostasis, whose disruption manifests as distinct clinical and neuropathological phenotypes. This mechanistic interpretation positions proteomic findings within a unified biochemical framework and underscores how differential weighting of shared molecular vulnerabilities may drive heterogeneity across atypical Parkinsonian syndromes [17,18,21].

Atypical Parkinsonisms, therefore, represent disorders of shared, interdependent biochemical systems, in which distinct clinical phenotypes emerge from differential engagement of common molecular vulnerabilities.

**Figure 1:**
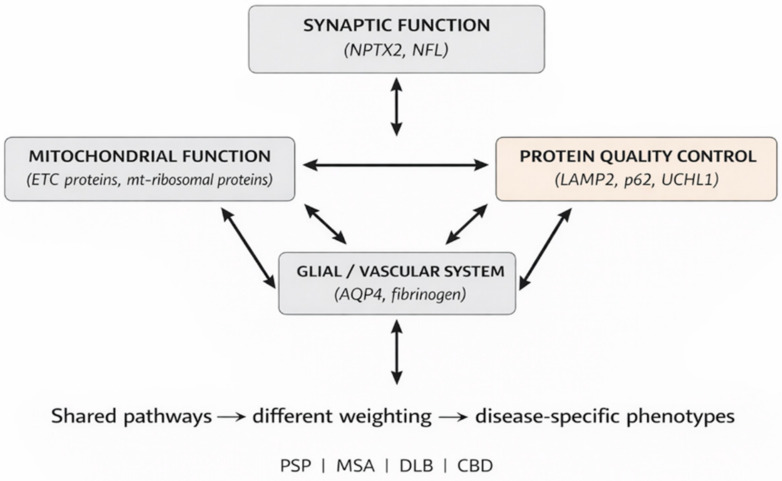
Schematic representation of interconnected biochemical systems involved in atypical Parkinsonisms. Proteomic alterations affect key cellular processes, including synaptic function, mitochondrial bioenergetics, protein quality control, and glial–vascular regulation. These systems form an integrated network rather than isolated disease-specific pathways. Differential involvement of shared molecular mechanisms contributes to disease-specific phenotypes across atypical Parkinsonian syndromes, including PSP, MSA, DLB, and CBD.

A schematic representation of these interconnected biochemical systems and their differential involvement across atypical Parkinsonian syndromes is shown in Figure 1.

Proteomic analysis in atypical Parkinsonian syndromes should be performed with cautious selection of sample sources, as analyses of different tissues or body fluids have shown inconsistent results. This could be partially explained by the leakproofness of the blood–brain barrier, preventing certain proteins from migrating to the serum. Diverse proteomic results highlight that most neurodegeneration-related processes take place in the central nervous system, not peripherally. The most accurate results are based on analysis of brain tissue samples; however, this is impossible in everyday clinical practice. Proteomic analysis could be useful in the evaluation of blood–brain barrier damage; fewer differences between the CSF and serum would indicate increasing leakiness. Therefore, in clinical practice, choosing samples for proteomic analysis should balance between invasiveness and the specimen’s laboratory value. Considering the primary localization of neurodegeneration-related processes, simultaneous proteomic analysis of CSF and serum represents a reasonable compromise. Additionally due to the fact that CBD is related to diverse clinical manifestations, extended proteomic evaluation in future studies would be valuable [28].

## 5. Conclusions

Proteomic analysis is a promising method of evaluation for atypical Parkinsonian syndromes, providing valuable insight into biochemical pathways connected to neurodegeneration; however, this method has several limitations. The results are strongly connected to the type of analyzed material; therefore, sample selection is crucial. Given that the data are limited and the exact mechanisms responsible for neurodegeneration remain unclear, it is almost impossible to establish whether a specific abnormality is a cause or effect of neurodegeneration. Data interpretation requires experience, as assessment of disrupted biochemical networks and pathways, not single protein concentrations, is needed.

The presented data suggest that proteomic evaluation of atypical Parkinsonisms may be a useful tool for further examination of the diseases; however, additional data based on larger groups of patients are required. Extended evaluation of the proteomic profiles of atypical Parkinsonisms may facilitate their differentiation and enable more efficient analysis of their pathogeneses, which have not been sufficiently explored [29,30,31]. Further analysis may provide crucial outcomes in the evaluation of subtypes of atypical Parkinsonisms [32].

## Figures and Tables

**Table 1:** Biochemical roles and mechanistic relevance of proteomic proteins.

Protein	Cellular Function	Biochemical Implication	Direction of Change	Disease Context	References
Neuronal pentraxin-2 (NPTX2)	Synaptic remodeling and AMPA receptor organization	Impaired synaptic plasticity and adaptability	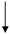	PSP	[17,26,27]
Neurofilament light/medium	Axonal cytoskeleton	Marker of axonal stress and transport dysfunction	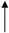	PSP, MSA, and DLB	[11,19,20]
Mitochondrial ETC proteins	Oxidative phosphorylation	Bioenergetic inefficiency and reduced ATP production	Altered	PSP	[17,18]
Mitochondrial ribosomal proteins	Mitochondrial translation	Impaired coordination of mitochondrial protein synthesis and OXPHOS	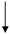	PSP	[17,18]
LAMP2	Autophagy–lysosome pathway	Reduced clearance of aggregation-prone proteins	Altered	PSP	[19]
p62/SQSTM1	Selective autophagy adaptor	Proteotoxic stress and impaired protein quality control	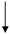	DLB and PDD	[24]
SLC7A11	Cystine/glutamate antiporter (system xc−)	Disturbed redox homeostasis and increased oxidative/excitotoxic vulnerability	Altered	DLB	[24]
Aquaporin-4	Astrocytic water transport	Impaired glymphatic clearance and extracellular homeostasis	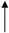	MSA	[17]
Fibrinogen	Coagulation/extracellular matrix	Neuroinflammatory amplification and BBB dysfunction	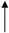	MSA, PSP, and DLB	[21,22]
UCHL1	Ubiquitin recycling	Reduced protein repair and proteome stability	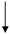	CBD	[23]

“Altered” indicates that the direction of change in expression was not consistently defined across studies or that it varied depending on the experimental context.

## Data Availability

The data is accessible on request.

## Abbreviations

CBD	Corticobasal Degeneration
DLB	Dementia with Lewy Bodies
MSA	Multiple System Atrophy
PDD	Parkinson’s Disease with Dementia
PSP	Progressive Supranuclear Palsy
